# The regional effect of spinal manipulation on the pressure pain threshold in asymptomatic subjects: a systematic literature review

**DOI:** 10.1186/s12998-018-0181-3

**Published:** 2018-04-19

**Authors:** Margaux Honoré, Charlotte Leboeuf-Yde, Olivier Gagey

**Affiliations:** 1 CIAMS, University of Paris-Sud, University of Paris-Saclay, F- 91405 Orsay Cedex, France; 2 CIAMS, University of Orléans, F- 45067 Orléans, France; 3 Institut Franco Européen de Chiropraxie, 24 boulevard Paul Vaillant Couturier, F- 94200 Ivry sur Seine, France

**Keywords:** Spinal manipulation, Experimental pain, Pressure pain threshold, Asymptomatic, Systematic review, Manipulation vertébrale, Douleur expérimentale, Seuil de douleur à la pression, Sujets asymptomatiques, Revue systématique

## Abstract

**Background:**

Spinal manipulation (SM) has been shown to have an effect on pain perception. More knowledge is needed on this phenomenon and it would be relevant to study its effect in asymptomatic subjects.

**Objectives:**

To compare regional effect of SM on pressure pain threshold (PPT) vs. sham, inactive control, mobilisation, another SM, and some type of physical therapy. In addition, we reported the results for the three different spinal regions.

**Method:**

A systematic search of literature was done using PubMed, Embase and Cochrane. Search terms were ((spinal manipulation) AND (experimental pain)); ((spinal manipulative therapy OR spinal manipulation) AND ((experimental pain OR quantitative sensory testing OR pressure pain threshold OR pain threshold)) (Final search: June 13th 2017). The inclusion criteria were SM performed anywhere in the spine; the use of PPT, PPT tested in an asymptomatic region and on the same day as the SM. Studies had to be experimental with at least one external or internal control group. Studies on only spinal motion or tenderness, other reviews, case reports, and less than 15 invited participants in each group were excluded. Evidence tables were constructed with information relevant to each research question and by spinal region. Results were reported in relation to statistical significance and were interpreted taking into account their quality.

**Results:**

Only 12 articles of 946 were accepted. The quality of studies was generally good. In 8 sham controlled studies, a psychologically and physiologically “credible” sham was found in only 2 studies. A significant difference was noted between SM vs. Sham, and between SM and an inactive control. No significant difference in PPT was found between SM and another SM, mobilisation or some type of physical therapy. The cervical region more often obtained significant findings as compared to studies in the thoracic or lumbar regions.

**Conclusion:**

SM has an effect regionally on pressure pain threshold in asymptomatic subjects. The clinical significance of this must be quantified. More knowledge is needed in relation to the comparison of different spinal regions and different types of interventions.

## RESUME

**But:**

Un effet de la manipulation vertébrale a été observé sur la perception de la douleur. Plus de connaissances sont nécessaires sur ce phénomène et il serait pertinent d’étudier cet effet sur les sujets asymptomatiques.

**Objectifs:**

Evaluer l’effet régional de la MV sur le seuil de douleur à la pression (SDP) comparé à un placébo, un groupe contrôle, la mobilisation, une autre MV, et un autre type de physiothérapie. De plus, nous avons rapporté les résultats pour les régions cervicale, dorsale et lombaire.

**Méthode:**

La revue systématique de la littérature a été faite en utilisant PubMed, Embase et Cochrane. Les termes de recherche étaient ((manipulation vertébrale) ET (douleur expérimentale)); ((thérapie manuelle spinale OU manipulation spinale) ET (douleur expérimentale OU test quantitatif sensoriel OU seuil de douleur à la pression OU seuil de douleur)). (La recherche finale date du 13 juin 2017). Les critères d’inclusion étaient MV exécutées à n’importe quel niveau de la colonne vertébrale; l’utilisation du SDP, testé dans une région asymptomatique et le même jour que la MV. Les études devaient être expérimentales avec au moins un groupe externe à la MV ou un groupe contrôle interne. Les études uniquement sur le mouvement de la colonne vertébrale, tensions des tissus paravertébraux, les autres revues, les études de cas et des études avec moins de 15 participants dans chaque groupe ont été exclus. Des tables d’évidence ont été construites avec les informations appropriées à chaque question de recherche et reportées par régions. Les résultats ont été rapportés avec leur signification statistique et ont été interprétés en tenant compte de leur qualité.

**Résultats:**

Seulement 12 articles sur 946 ont été acceptés. La qualité des études est. en général bonne. Sur les 8 études contrôlées par placébo, un placébo psychologiquement et physiologiquement « crédible » a été trouvé seulement dans 2 études. Une différence significative a été notée entre MV et le placébo, et entre MV et contrôle inactif. Aucune différence significative dans SDP n’a été trouvée entre MV et une mobilisation, une autre MV, ou un autre type de physiothérapie. Des résultats significatifs ont été trouvés plus souvent dans la région cervicale comparé aux études aux niveaux thoraciques et lombaires.

**Conclusion:**

La manipulation vertébrale a un effet régional sur le seuil de la douleur à la pression chez des sujets asymptomatiques. Sa différence clinique doit être quantifiée. Plus de connaissance est. nécessaire à propos de la comparaison des différentes régions spinales et différents types d’interventions.

## Background

Spinal manipulation (SM) is a therapeutic tool used by several health care professions [1]. It is reasonably inexpensive [2] and recognized to have a clinically significant impact on musculoskeletal pain [3]. Nevertheless, its indications and mechanisms of action are still not well established for example in relation to pain reduction.

### Spinal manipulation

#### Definition

SM can be performed manually, resulting in a passive high velocity low amplitude movement that separates the vertebral joint surfaces [4]. Various positions of the therapist and angles of his hands can be used to obtain this result. Regardless, it is often accompanied by a cracking sensation and sound [5]. SM takes the targeted joint beyond its passive amplitude without causing an anatomical lesion [6]. It is a mechanical event that seems to decrease briefly the intra-discal pressure and to stretch the surrounding muscles, thus causing their relaxation [7]. It can be differentiated from mobilisation, where the movement would be one or several low velocity low amplitude actions on the joints by the therapist, usually not accompanied by the “crack”.

SM can also be mechanically assisted with an instrument called “activator”. It can be set to produce a high velocity low amplitude force directly over a joint, just as the classical manual type of SM. In researchon SM, the activator is often used as a sham procedure, with the instrument set on zero force, but still producing the same sound as when at normal force.

These two types of SM are traditionally used as interchangeable by chiropractors, although they may of course have different mechanisms. For the purpose of this review, we shall consider both of them as equally representative of SM.

#### Impact on clinical pain

Patients often report immediate improvement of pain after SM. For example, 63% of 984 patients with low back pain reported immediate improvement in a multi-center study [8], and according to the results of a meta-analysis, SM has better outcome compared with exercises, education of the patient, and other forms of manual therapies (physiotherapy) in relation to experimentally induced pain [9]. This hypoalgesic effect is the object of several theories and models, and has been tested using experimentally induced pain.

### Spinal manipulation and experimental pain

#### Pressure pain threshold

To examine this hypoalgesic effect, studies have used experimentally induced pain on asymptomatic subjects, to avoid the presence of co-morbidity and/or a central pain inhibitory effect likely to be present with ongoing pain.

The perception of pain is a complex phenomenon that can be induced by various external stimuli, such as pressure or temperature. A systematic review of the literature on asymptomatic healthy subjects published in 2012 showed that SM has an effect on pain induced by pressure, by increasing the pain threshold. It would seem that SM acts more on the pain produced by pressure than by temperature [10]. Pain on pressure is often tested via the pressure pain threshold (PPT), which is defined as the minimal pressure which provokes a pain or a discomfort [11]. It has the advantage to be objective and valid, with a good reliability [12]. It is present in all individuals and, thus, possible to be estimated for everybody. The PPT can be relatively easily determined by the means of an algometer, a device making it possible to measure exactly the applied pressure and in a precise zone.

#### Concerning the regional testing

Although many studies report an immediate effect on pain perception following SM [10], the literature seems undecided as to whether this effect is purely local or if it extends also into other anatomical areas. According to a systematic critical review of the effect of SM on asymptomatic tissues, local and regional effects were often reported. However, all studies reporting a distant effect failed to use a blinded design [10]. The differentiation between “local” and “regional” is not always clear, but it seems likely that any pain-reducing effect would follow the nerve supply of the manipulated segment rather than occurring only directly in the manipulated area.

### Spinal manipulation and experimental groups

#### Sham procedure and inactive control

To determine the effect of SM on experimental pain, SM must be compared to a sham procedure, or possibly an inactive control. A sham procedure is used in order to induce a placebo effect, with the intent to “fool” the subjects and to make them believe they received an active treatment. As it is relatively easy for study subjects to differentiate between a real SM and various attempted sham maneuvers, the subjects should be as naïve to SM as possible. An inactive control is when the subjects are resting, and nothing is done to them. However, in such a situation, study subjects are well aware of not being treated, so it is not comparable to a successful sham procedure. If there would be no physiological effect of SM (i.e. no increased PPT), one would expect there to be no difference in PPT readings between SM and a successful sham. However, an unsuccessful sham treatment and a control treatment would be expected to result in higher PPT values for the SM group, as the study subjects would be likely to realize that this is the active procedure, which could result in a placebo effect. Therefore, it is relevant to compare the results in studies with credible shams vs. those without and also, results in purely passive control groups.

#### *Spinal manipulation* vs. *mobilisation*

Results from a systematic review on healthy subjects indicated that there was no difference in results between studies that concentrated on the crack and those that did not [10]. Similarly and perhaps for this reason, there is a doubt concerning the physiological effect of SM compared to mobilisation, and one study in the systematic review even found that mobilisation had a stronger effect than SM [13]. Therefore, it would be relevant to investigate also the difference of outcome between SM and mobilisation.

#### *Spinal manipulation* vs. *other spinal manipulation*

The literature indicates that SM may be more effective in certain regions than others, without giving a clear answer [10], for which reason a comparison between SM in different spinal regions is relevant as well.

#### *Spinal manipulation* vs. *some type of physical therapy*

A recent meta-analysis showed a significant “effect” of SM on the PPT, as compared to other interventions, such as exercise, education of the patient, and physiotherapy [9], but the difference is rather small, making also this an interesting topic of investigation.

### Aims and search questions

Because of the gaps in our understanding of the effect of SM as compared to sham and other types of treatments, and because more articles have been published on this topic since 2012, it would be timely to update the previous review and enlarge our scope of approach. Therefore, in this systematic literature review we investigated if SM influences the PPT in symptomatic subjects, i.e. in subjects without symptoms in the tested area. A “tested area” was defined as the immediate anatomical site (i.e within the dermatome) where SM was given and PPT testing took place. In addition to the previous survey, we compared the outcome between SM and different comparison groups. We had five specific research questions in relation to the regional effect on the PPT after SM.

#### Research questions 1–2

Is there a statistically significant effect on the PPT levels when comparing (i) SM to sham, and/or (i) SM to an inactive control?

#### Research questions 3–5

Are there any statistically significant differences of outcomes in the PPT when SM is compared to (i) mobilisation, (ii) another type of SM or SM in another spinal region, and/or (iii) some type of physical therapy?

In addition, we noted the results for the different spinal regions.

## Method

### Design

This systematic critical review consists of an update and extension of previous work on almost the same topic [10], and was registered in Prospero on February 27, 2017 (receipt [58207]).

### Search strategy

We did a systematical literature search of PubMed, Embase and Cochrane using the search terms [(spinal manipulation) **AND** (experimental pain)]; [(spinal manipulative therapy **OR** spinal manipulation) **AND** ((experimental pain **OR** quantitative sensory testing **OR** pressure pain threshold **OR** pain threshold)]. We generally used the same search terms as the previous systematic review conducted on the same topic [10]. We also searched reference lists of relevant articles including those in the previous review [10]. The present search was performed between 2011.01.01 and 2017.06.13.

**Fig. 1:**
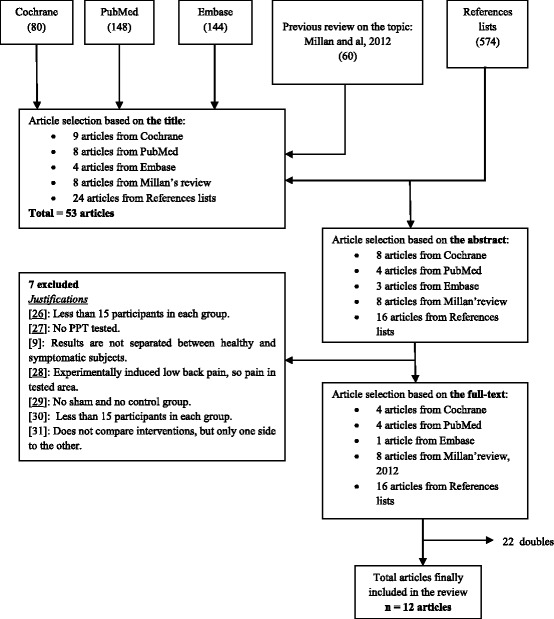
Flow Chart showing the selection process of a systematic review on spinal manipulation and pressure pain threshold [9, 10, 26–31]

In the screening process, our inclusion criteria were SM performed manually and/or mechanically assisted, anywhere in the spine; the use of PPT, in an asymptomatic region and on the same day as SM, reported in experimental studies with at least one external or internal control group. Studies on only spinal motion or tenderness, other reviews, case reports and studies with less than 15 invited participants in each group were excluded. We used the preferred reporting item for systematic reviews and meta-analysis (PRISMA) flow chart (Fig. 1) to record our screening of the articles for inclusion and to report the flow of the review.

### Extraction of data

We created our own evidence checklists, based on concepts described in CONSORT statements and the previous review [10]. All items were extracted from the Method and Results sections only.

The extraction of data was done blindly by the first author and one supervisor with extensive experience in systematic reviews. Both were chiropractors and were able to interpret the information in articles in relation to how SM and sham treatments were performed. Then, they compared the data and, if there were any disagreements, a third person could intervene and decide. Clear definitions and the rationale for the descriptive and the quality checklists items have been described in Tables 1 and 2. Results were reported in Table 3 and Table 4. Finally, the outcomes were noted for each article as significant or not significant (Table 5); exact estimates and effect size to be reported elsewhere.

**Table 1:** Description and interpretation of items used in a systematic review on spinal manipulation and pressure pain threshold

Descriptive items	Design	Sham Control Comparison with other treatment	Source of study sample	Age of participants Mean (range)	N subjects 1/invited 2/final analysis 3) per treatment group 4) (M/F)	Intervention groups: Types and area	Treatment area - “Lesion” - Standard	Pain measured where in relation to SM? Regional Remote	PPT measured when?
Interpretation	Randomized controlled trial and/or cross over studies	Description of the group of comparison	General population, volunteers, students, to estimate if they were naïve		Describes size of study and indicates if there were losses or exclusions during the study.	Treatment group was indicated in relation to area of spine manipulated and comparison groups were described as Sham and Control with an indication of the area where the sham was given	“Lesion”: The treating clinicians decides where to apply the SM based on clinical examination. “Standard”: All study subjects receive the SM in a predetermined area	“Regional” was defined as an area within the dermatome of the area of SM “Remote” was defined as an area outside this dermatome	PPT could be measured at base-line and at various time intervals after the intervention

M/F: number of Males and Females in the study/SM: Spinal Manipulation/PPT: Pressure Pain Threshold

**Table 2:** Quality items, rationale for inclusion in quality assessment, interpretation and scoring used in a systematic review on spinal manipulation and pressure pain threshold

Quality items	Description of the random allocation: 1)Randomization method 2)Concealment	Treatment performed by appropriate and experienced person?	Is the intervention described? 1. SM 2. Sham 3.Comparison 4. Control	Is the assessment blinded? 1. Assessor/intervention 2.Statistician/intervention	The sham procedure: (Yes/No/NA) 1.Naïve subjects 2. In the same position as SM? 3. Assessed Conclusion: Is the sham psychologically acceptable (1 pt), possibly acceptable (0.5 pt), not acceptable (0 pt)	If no sham procedure, at least are the subjects naïve? (Yes/No/NA)	Is the measurement procedure described?	Is reliability of the outcome variables reported?	Were pain readings taken more than once at each point?	After the study started, are losses and exclusions of study subjects reported or evident?
Rationale for inclusion in quality assessment	1)Ensuring equal distribution of study subjects 2) Prevent risk of cheating during group allocation and risk of bias during assessment	Ensuring interventions are appropriately administered	Ensuring that study can be reproduced	1Preventing risk of assessor bias 2.Preventing “data massage”	Assuring the credibility of the sham both from a psychological and physiological aspect	Ensuring that there is no risk of participant bias	Ensuring that study can be reproduced	As validity is difficult to obtain ensuring that, at least, the outcome variable is reliable	More than one reading is needed to avoid unrepresentative data	Making it possible to detect risk of exclusion/attrition bias
Interpretation details (where relevant)	1).as it said that participants were allocated into groups in a random fashion? 2) Was it stated that groups were concealed during random allocation?	Appropriate: practitioner with training in SM We believed the study when authors reported some sort of experience or expertise for the treating clinicians	If we understood what had been done in the experiment, we considered this acceptable	This had to be stated in the text	1–3. A sham procedure may be able to “fool” a study subject but…	This had to be stated in the text	If we understood the procedure, we considered this acceptable	This could be reported with a reference to previous study or a reliability study could be reported in the Result section	This had to be stated in the Method or Result section	This had to be stated or obvious from information given in Tables or Result section
Scoring	1. 0.5 pt. 2. 0.5 pt	1 pt	1 pt	1. 0.5 pt. 2. 0.5 pt	1 or 2: 0.5 pt. 1 + 2: 1 pt. 3 found acceptable: 1 pt. 3 not found acceptable: 0 pt. Conclusion: Is the sham psychologically acceptable (when 1 pt. is given), possibly acceptable (when 0.5 pt. is given), not acceptable (when 0 pt. is given)	Yes: 1 pt. No: 0 pt. If NA, this case is not taken into account.	1 pt	1 pt	1 pt	1 pt

*SM* Spinal Manipulation, *NA* Not Applicable

**Table 3:** Description of 12 studies included in a systematic review on spinal manipulation and pressure pain threshold

1st author Year Country [ref #]	Design	Sham Control Comparison with other treatment	Source of study sample	Age of participants Mean (range)	N subjects 1/invited 2/final analysis 3) per treatment group 4) (M/F)	Intervention groups: Types and area	Treatment area - “Lesion” - Standard	Pain measured where in relation to SM? Regional Remote	PPT measured when?
Fryer 2004 Australia [21]	RCT	Sham Comparison	Students	- (19–34)	1) 96 2) - 3) 3 × 32 4) 39/57	1) SM thoracic 2) Thoracic Mobilization 3) Sham laser	Lesion	Regional	Before and after SM
Ruiz-Saez 2007 Spain [16]	RCT	Sham	Volunteers, general population with palpatory pain in trapezius	31 (19–45)	1) 72 2) 72 3) 2 × 36 4) 26/46	1) Cervical SM 2) Sham cervical	Lesion	Regional	Before and 1,5 and 10 min after SM
Fernandez de las Penas 2007 Spain [19]	RCT cross over	Sham Control Comparison	Students in physical therapy, occupational therapy, rehabilitation, and physical medicine	21 (19–25)	1) 15 2) 15 3) 7 for Right SM 8 for Left SM 4) 7/8	1) Cervical SM on the right or left 2) Sham cervical 3) Control	Standard	Regional	Before and 5 min after SM
Hamilton 2007 Australia [20]	RCT	Sham Comparison	Students and teaching body of university	23 (−)	1) 90 2) - 3) 35;25;30 4) 29/61	1) SM cervical 2) Sham thoracic 3) Muscle energy technique (suboccipital and trapezius muscles)	Standard	Regional	Before 5 and 30 min after SM
Fernandez de las Penas 2008 Spain [18]	RCT	Sham Comparison	Volunteers from general population	26 (19–35)	1) 30 2) 30 3) 3 × 10 4) 13/17	1) Cervico-thoracic SM (dominant side) 2) Cervico-thoracic SM (non-dominant side) 3) Sham cervical	Standard	Regional	Before and 5 min after SM
Thomson 2009 Sweden [13]	RCT	Sham Comparison	Osteopathic students and teachers	27	1) 50 2) 50 3) 18;19;13 4) 29/21	1)Lumbar SM 2) Lumbar mobilization 3) Sham lumbar laser	Lesion	Regional	Before and immediately after SM
Oliveira Campelo 2010 Spain [22]	RCT	Control Comparison	Volunteers from school of technology with palpatory pain in jaw muscle	20 (18–30)	1) - 2) 122 3) 40; 41; 41 4) 31/91	1) Cervical SM 2) Soft tissue 3) Control	Standard	Regional	Before and 2 min after SM
Bishop 2011 USA [24]	Randomized experimental design	Control Comparison	Volunteers recruited by posters	23 (−)	1) 90 2) - 3) 3 × 30 4) 24/66	1) SM Cervico-Thoracic region 2) Cervical exercises 3) Control	Standard	Regional and remote	Before and immediately after SM
Yu 2012 China [15]	RCT cross-over	Sham	General population and medical students	24 (−)	1) 30 2) 30 3) 30 × 2 4) 19/11	1) SM lumbar (activator) 2) Sham (detuned activator)	Lesion using activator protocol	Regional and remote	Before and after SM
Srbely 2013 Canada [17]	RCT	Sham	Volunteer university students	29 for treatment 27 for sham (−)	1) 44 2) 33 3) 18 for SM and 18 for sham 4)19/17	1) SM cervical 2) Sham cervical	Standard	Regional and remote	Before, 1,5,10 and 15 min after SM
Jordon 2016 USA [23]	RCT	Control Comparison	University community	22 (18–32)	1) 57 2) 54 3) 18;19;17 4) 25/32	1) Rest 2) Lumbar + Cervical SM 3) Cervical + Lumbar SM	Standard	Regional and remote	Before and after SM
Alonso Perez 2016 Spain [22]	RCT	Comparisons	University population	29 (−)	1) 83 2) 74 3) 25;24;25 4) 39/36	1) Cervical SM 2) Cervical mobilization 3) Cervical glide mobilization	Standard	Regional	Before and after SM

*RCT* randomized controlled trial, *SM* spinal manipulation

**Table 4:** Quality items and scores of 12 studies included in a systematic review on spinal manipulation and pressure pain threshold

First author Year Country [ref #]	Is there a description of the random allocation? 1)Randomization method 2) Concealment	Is treatment performed by experienced person?	Is the intervention described? 1. SM 2. Sham 3. Comparison 4. Control	Is the assessment blinded? 1.Assessor/intervention 2.statistician/intervention	The sham procedure: (Yes/No/NA) 1.Naïve subjects 2. In the same position as SM? 3. Assessed Conclusion: Is the sham psychologically acceptable (1 pt), possibly acceptable (0.5 pt), not acceptable (0 pt)	If comparison between interventions are the subjects naïve? (Yes/No/NA)	Is the measurement procedure described?	Is reliability of the outcome variables reported?	Were pain readings taken more than once at each point?	After the study started, are losses and exclusions of study subjects reported or evident?	Score for sham studies	Score for comparison studies
Fryer 2004 Australia [21]	1) Yes 2) No	No	1) Yes 2) Yes 3) Yes 4) NA	1) Yes 2) No	1. No 2. No 3. No	No	Yes	Yes	Yes	No		
					Conclusion: Not acceptable							
	0.5	0	1	0.5	0	0	1	1	1	0	5/9	5/9
Ruiz-Saez 2007 Spain [16]	1) Yes 2) No	Yes	1) Yes 2) Yes 3) NA 4) NA	1) Yes 2) No	1. Yes 2. Yes 3. No	NA	Yes	Yes	Yes	Yes		
					Conclusion: Acceptable							
	0.5	1	1	0.5			1	1	1	1		
					1						8/9	NA
Fernandez de las Penas 2007 Spain [19]	1) No 2) No	Yes	1) Yes 2) Yes 3) Yes 4) Yes	1) Yes 2) No	1. Yes 2. Yes 3. No	Yes	Yes	Yes	Yes	No		
					Conclusion: Acceptable							
	0	1	1	0.5	1	1	1	1	1	0	6,5/9	6.5/9
Hamilton 2007 Australia [20]	1) Yes 2) No	Yes	1) Yes 2) Yes 3) Yes 4) NA	1) Yes 2) No	1. Yes 2. No 3. No	Yes	Yes	Yes	Yes	No		
					Conclusion: Possibly acceptable							
	0.5	1	1	0.5	0.5	1	1	1	1	0	6,5/9	7/9
Fernandez de las Penas 2008 Spain [18]	1) Yes 2) No	Yes	1) Yes 2) Yes 3) Yes 4) Yes	1) Yes 2) No	1. Yes 2. Yes 3. No	Yes	Yes	Yes	Yes	No		
					Conclusion: Acceptable							
	0.5	1	1	0.5	1	1	1	1	1			
										0	7/9	7/9
Thomson 2009 Sweden [13]	1) Yes 2) No	Yes	1) Yes 2) Yes 3) Yes 4) NA	1) Yes 2) No	1. No 2. No 3. No	No	Yes	Yes	Yes	No		
					Conclusion: Not acceptable							
	0.5	1	1	0.5	0	0	1	1	1			
										0	6/9	6/9
Oliveira Campelo 2010 Spain [22]	1) Yes 2) No	Yes	1) Yes 2) NA 3) Yes 4) Yes	1) Yes 2) No	NA	Yes	Yes	Yes	Yes	Yes		
	0.5	1	1	0.5		1	1	1	1	1		
											NA	8/9
Bishop 2011 USA [24]	1) Yes 2) No	No	1) Yes 2) NA 3) Yes 4) Yes	1) No 2) No	NA	Yes	Yes	No	No	No		
	0.5	0	1	0		1	1	0	0			
										0	NA	3.5/9
Yu 2012 China [15]	1) Yes 2) No	Yes	1) Yes 2) Yes 3) NA 3) NA	1) Yes 2) No	1. Yes 2. Yes 3. No	NA	Yes	Yes	Yes	Yes		
					Conclusion: Acceptable							
	0.5	1	1	0.5	1		1	1	1	1	8/9	NA
Srbely 2013 Canada [17]	1) Yes 2) Yes	Yes	1) Yes 2) Yes 3) NA 4) NA	1) Yes 2) No	1. Yes 2. Yes 3. No	NA	Yes	No	Yes	Yes		
					No Conclusion: Acceptable							
	1	1	1	0.5			1	0	1			
					1					1	7,5/9	NA
Jordon 2016 USA [23]	1) Yes 2) Yes	Yes	1) Yes 2) NA 3) Yes 4) Yes	1) Yes 2) No	NA	Yes	Yes	Yes	No	Yes		
	1	1	1	0.5		1	1	1	0			
										1	NA	7.5/9
Alonso Perez 2016 Spain [22]	1) Yes 2) Yes	Yes	1) Yes 2) NA 3) Yes 4) NA	1) Yes 2) No	NA	Yes	Yes	Yes	Yes	Yes		
	1	1	1	0.5		1	1	1	1			
										1	NA	8.5/9

*NA* Not applicable, *SM* Spinal manipulation

**Table 5:** Results from 12 studies included in a systematic review on spinal manipulation and pressure pain threshold

First author Year Country [#ref]	Area of SM	Regional: (…………….)	Sign. diff. SM vs. Sham? Was the sham credible? (Yes/No)	Sign. diff SM vs. Control	Sign. diff. SM vs. Other SM (……………)	Sign. diff. SM vs. Mobilization. (………………)	Sign. diff SM vs. Other therapy (…………….)	Quality Score (for sham and comparison groups)
Srbely 2013 Canada [17]	Cervical (bilateral)	Regional: (infraspinatus muscle)	Regional: Yes Sham procedure: Yes					7.5/9 for sham
Yu 2012 China [15]	Lumbar	Regional: - (L5-S1 over apophyseal joints) - (L5 dermatome)	Regional: Yes Sham procedure: Yes					8/9 for sham
Thomson 2009 Sweden [13]	Lumbar	Regional: (Spinous process of L3)	Regional: No Sham procedure: No			No (Mobilization lumbar spine)		6/9for sham 6/9 for comparison
Fernandez de las Penas 2008 Spain [18]	Cervical	Regional: (C5-C6 level at dominant and non-dominant side)	Regional: Yes Sham procedure: No		No (SMT dominant side vs. non-dominant side)			7/9for sham 7/9 for comparison
Ruiz Saez 2007 Spain [16]	Cervical	Regional: (Upper trapezius latent trigger points)	Regional: Yes Sham procedure: No					8/9 for sham
Fernandez de las Penas 2007 Spain [19]	Cervical	Regional: (Ipsilateral and contralateral epicondyle)	Regional: Yes Sham procedure: No	Yes	Yes			6.5/9 for sham 6.5/9 for comparison
Fryer 2004 Australia [21]	Thoracic	Regional: (Thoracic spinous process between T1 and T4)	Regional: No Sham procedure: No			No (Extension mobilization of thoracic spine)		5/9 for sham 5/9 for comparison
Bishop 2011 USA [24]	Thoracic	Regional: (Between first and second fingers)		Regional: No Remote: No			No (Exercise)	3.5/9 for comparison
Oliveira Campelo 2010 Spain [22]	Cervical	Regional: (Masseter and temporalis latent trigger points)		Regional: - Masseter: Yes - Temporalis:Yes				8/9 for comparison
Hamilton 2007 Australia [20]	Cervical	Regional: (Between C2 and C0)	Regional: No Sham procedure: No				No (Muscle energy technique)	6.5/9 for sham 7/9 for comparison
Jordon 2016 USA [23]	Cervical and Lumbar	Regional: (Lateral epicondyle of humerus and upper trapezius bilaterally)			Regional: No			7.5/9 for comparison
Alonso Perez 2016 Spain [22]	Cervical	Regional: (Cervical process of C7, bilateral Trapezius muscle, epicondyle region)			Regional: No between groups results but within groups results presented			8.5/9 for comparison

### Classifying articles by their quality

The selected articles were checked for each quality item. We took a special interest in the sham procedure and determined two specific approaches, investigating if the sham was both psychologically and physiologically credible. For the psychological aspect, the study had to include naïve subjects and the sham should resemble the intervention, and/or it had to be assessed and found to “work”, with the use of a post-study questionnaire.

For the physiological approach, we expected there to be some type of tension, but not directly over the spinal column, to mimic closely the real intervention. It would be associated with a low velocity low amplitude sham “thrust”, to imitate the same mechanical components of a manual SM but without a direct action on the spinal column [14]. This type of procedure has been shown to confuse study participants and to do so over a whole treatment program [14]. For the mechanically assisted SM, the same characteristics as with the “active” (activator) treatment are expected, but with the activator set with no force at all and producing the same sound as the real intervention. In conclusion, the sham would be considered completely “credible” only if both aspects (psychological and physiological) were fulfilled.

Thereafter, we created a quality score, by giving one point for each fulfilled item. This score was arbitrarily divided into “high”, “medium” and “low”, to indicate the general quality of the articles (Table 6). Articles were listed in descending order based on the quality score in the result checklists. For the sham studies, we also separated them into “sham credible” and “sham not credible”.

**Table 6:** Summary of quality scores and quality classification for 12 articles included in a systematic review on spinal manipulation and pressure pain threshold

	First Author, Year [ref]	Score	Quality scale
SHAM STUDIES	Yu, 2012 [15]; Ruiz Saez, 2007 [16]	8/9	High
	Srbely, 2013[17]	7.5/9	High
	Fernadez de la Penas, 2008 *[18]	7/9	Medium
	Fernadez de la Penas, 2007 [19]; Hamilton [20]	6.5/9	Medium
	Thomson, 2009 [13]	6/9	Medium
	Fryer, 2004 [21]	5/9	Medium
COMPARISON STUDIES	Alonso Peres, 2016 [22]	8.5/9	High
	Oliveira Campelo, 2010 [23]	8/9	High
	Jordon, 2016 [24]	7.5/9	High
	Hamilton, 2007 [20]; Fernandez de las Penas, 2008 [18]	7/9	High
	Fernadez de la Penas, 2007 [19]	6.5/9	Medium
	Thomson, 2009 [13]	6/9	Medium
	Fryer, 2004 [21]	5/9	Medium
	Bishop, 2011 [25]	3.5/9	Low

Classification: Low: 0–4.5pts; Medium: 5–6.5pts; High: 7-9pts. *Some articles were listed twice in the table, as they used a sham procedure and compared a spinal manipulation to another intervention

### Data synthesis

The evidence tables were used in a systematic fashion to obtain answers to our research questions, by taking into account the general quality of the articles and in the case of the sham studies, the credibility of the sham procedure in relation to the psychological and physiological aspects, both independently and in combination.

## Results

In all, 148 potentially relevant articles titles were found in the PubMed search, 80 in Cochrane, 144 in Embase, and 60 were selected from the previous review on the same topic. At the end of the screening process, the hand search of the reference lists of all selected articles resulted in 574 potential articles. Only 12 articles were finally selected. Please see Fig. 1.

### Description of studies

As it can be seen in Table 7, eight articles reported on SM vs. sham, with five articles measuring the PPT in the cervical, one in the thoracic, and two in the lumbar region. Only three articles tested SM vs. inactive control, with two articles in the cervical region, and one in the thoracic region. Two articles were found testing SM vs. SM, one in the cervical region and the other for both the cervical and lumbar regions. We found three articles on SM vs. mobilisation, one in the cervical region, one in the thoracic region, and one in the lumbar region; and three articles on SM vs. some type of physical therapy, two in the cervical region and one in the thoracic region.

**Table 7:** Significant difference of outcomes between-groups in 8 studies that compared a spinal manipulation to a sham procedure, including information on the sham and areas tested included in a systematic review on spinal manipulation and pressure pain threshold

Regions	First author, year [ref]	Psychologically acceptable sham	Physiologically acceptable sham	In conclusion, was the sham completely credible? (Yes/No)	General quality classification of articles	Significant difference of outcomes between groups (Yes/No)
Cervical	Ruiz-Saez, 2007 [16]	Acceptable	Not acceptable	No	High	Yes
	Srbely, 2013 [17]	Acceptable	Acceptable	Yes	High	Yes
	Fernandez de las Penas, 2008 [18]	Acceptable	Not acceptable	No	Medium	Yes
	Fernandez de la Penas, 2007 [19]	Acceptable	Not acceptable	No	Medium	Yes
	Hamilton, 2007 [20]	Possibly acceptable	Not acceptable	No	Medium	No
Lumbar	Yu, 2012 [15]	Acceptable	Acceptable	Yes	High	Yes
	Thompson, 2009 [13]	Not acceptable	Not acceptable	No	Medium	No
Thoracic	Fryer, 2004 [21]	Not acceptable	Not acceptable	No	Medium	No

The level of quality was generally medium to high, except for one article that we considered to be of low quality (Table 4). The sham procedure was found psychologically acceptable for six articles ([15–20]), physiologically acceptable for two articles ([15, 17]) and both aspects were acceptable for two articles ([15, 17]), thus classified by us as completely “credible”. Only two were classified by us to be unacceptable both psychologically and physiologically (Table 7).

Examples of methodological weaknesses were that none of the twelve studies reported a detailed definition of the statistical imputation method, or reported on the blindness of the statistician, and none assessed the credibility of the placebo procedure with a post-study questionnaire. Only half of the studies reported on losses and exclusions of study subjects in their experiments.

### Spinal manipulation vs. sham

#### *Significant difference in PPT readings*

Out of eight articles on SM vs. sham, all of high to medium quality, two were of us considered completely credible, and both of these reported a significant effect on the PPT. Another three were considered only psychologically credible, and three of these reported a significant difference in favor of the treatment group. The three studies with a sham considered by us to be unsuccessful in relation to both aspects did not find such differences (Table 7).

#### *Difference between regions*

Four of the five studies that measured the PPT in the cervical spine had positive findings. One out of two studies measuring outcome in the lumbar spine reported a significant difference, whereas the only study to concentrate on the thoracic spine had no significant findings. For further information relating to general quality and credibility of sham, please see Table 7.

### Spinal manipulation vs. inactive control

#### *Significant difference in PPT readings*

Out of the three articles that addressed this matter, two of high and medium quality, reported a significant difference in PPT readings whereas the third article, considered not to be of good quality, found no significant difference. See Table 8.

**Table 8:** Significant difference of outcomes between -groups of 3 studies that compared a spinal manipulation to an inactive control included in a systematic review on spinal manipulation and pressure pain threshold

Regions	First Author, Year [ref]	General quality classification of articles	Significant difference of outcomes between groups (Yes/No)
Cervical	Fernandez de la Penas, 2007 [19]	Medium	Yes
	Oliveira Campelo, 2010 [22]	High	Yes
Thoracic	Bishop, 2011 [25]	Low	No

#### *Difference between regions*

The two studies on the cervical spine reported a significant difference, whereas the study on the thoracic spine did not (Table 8).

### **Spinal manipulation vs. mobilisation**

#### *Significant difference in PPT readings*

Three studies compared SM vs. mobilisation, all of medium to high quality. One did not report between-group results and the other two found no significant difference. See Table 9.

**Table 9:** Significant difference of outcomes between- groups of 3 studies that compared a spinal manipulation to a mobilisation included in a systematic review on spinal manipulation and pressure pain threshold

Regions	First Author, Year [ref]	General quality classification of articles	Significant difference of outcomes between groups (Yes/No)
Cervical	Alonso Peres, 2016 [22]	High	*No between groups results*
Thoracic	Fryer, 2004 [21]	Medium	No
Lumbar	Thompson, 2009 [13]	Medium	No

#### *Difference between regions*

The thoracic and lumbar regions were investigated in respectively two studies, none of which detected a significant difference (Table 9).

### Spinal manipulation vs. another spinal manipulation

#### *Significant difference in PPT readings*

Two articles investigated SM performed in different parts of the spine, both of high quality. No difference was found. None compared different techniques. See Table 10.

**Table 10:** Significant difference of outcomes between- groups of 2 studies that compared a spinal manipulation to another spinal manipulation included in a systematic review on spinal manipulation and pressure pain threshold

Regions	First author, Year [ref]	General quality classification of articles	Significant difference of outcomes between groups (Yes/No)
Cervical	Fernandez de la Penas,2008 [18]	High	No
Cervical and Lumbar	Jordon, 2016 [23]	High	No

#### *Difference between regions*

Two studies on the cervical spine found no difference in outcomes between areas of treatment. The third study investigated if the order of treatment (neck before or after low back) affected the outcome, which it did not (Table 10).

### Spinal manipulation vs. some type of physical therapy

#### *Significant difference in PPT readings*

Three articles reported on the comparison of SM vs. some type of physical therapy; two were of high quality and one was not. One article had contradictory results, both positive and absence of positive findings, whereas the others found no significant difference. See Table 11.

**Table 11:** Significant difference of outcomes between- groups of 3 studies that compared a spinal manipulation to some type of physical therapy included in a systematic review on spinal manipulation and pressure pain threshold

Regions	First Author, Year [ref]	General quality classification of articles	Significant difference of outcomes between groups (Yes/No)	
Cervical	Hamilton, 2007 [20]	High	No	
	Oliveira Campelo, 2010 [22]	High	Yes: Masseter	No: Temporalis
Thoracic	Bishop, 2011 [25]	Low	No	

#### *Difference between regions*

Two studies reported on the cervical spine and one on the thoracic region. Only one of the cervical studies reported some significant findings (Table 11).

## Discussion

### Summary

This systematic review consisting of 12 relevant articles studied the regional effect of SM on PPT in asymptomatic subjects. Such an effect was found when SM was compared to a sham and an inactive control, but there were generally no difference in outcome when compared to mobilisation, another SM, or some type of physical therapy. In relation to the region, the majority of the studies of the cervical region obtained significant findings as compared to studies on the other regions. Our review consists of four additional studies on PPT as compared to two systematic reviews from 2012 [9, 10], in which eight articles on PPT were identical to ours. Our findings were similar to their conclusions that SM under experimental conditions has an effect on PPT.

### Explanations

It would not be surprising that a treatment, in comparison with an inactive control, results in better results, as the study subjects in the control group would be able to guess that they did not receive an active treatment. Interestingly, we found that when the sham was credible, the results were more likely to be significant than when the sham lacked credibility. This finding, therefore, contradicts the placebo effect, which would have been expected to occur rather in studies with poorly concealed inactive treatments. Therefore, our findings point in the direction of a true effect of SM on PPT in asymptomatic subjects.

When SM was compared to other types of intervention, such as mobilisation, SM in another area, and some type of physical therapy, there was no significant difference, indicating that they probably all have an “effect”, as they all increased the PPT levels on asymptomatic subjects.

Our results raise the question, as to whether the cervical spine is more sensitive to this type of experimentation. This could be explained by the possibility that mechanoreceptors are distributed unevenly through the body, and that the cervical spine tends to have more of them than the thoracic and lumbar spine. However, in order to investigate if there is a true difference between spinal regions, specific studies will have to be designed that specifically test several areas. In this review, no such studies were found.

### Methodological considerations of our own review

We created our own checklists to answer our research questions, but we did follow the CONSORT statements and the previous review on the same topic [10]. We kept searching the main sites (PubMed, Embase, and Cochrane) regularly to capture resent publications. However, our university did not have access to all search databases, so it is possible that we missed out on some studies.

Both readers were blind during the process of selecting articles and extracting data in the checklists. In case of a disagreement, a third reviewer could intervene, but this was never needed, indicating that our procedure was user friendly.

In our review, one of the quality criteria was that PPT measurements should have been made at least twice at each anatomical region. However, it may well be possible that this is in fact not necessary [32].

Various types of SM and mobilisation were used and they could, theoretically, have different modes of action, which was not taken into account in this analysis, nor do other authors seem to have dealt with this issue. The same could be argued for different sham procedures, as these were not identical between studies, and different approaches could have different effects.

The concept of the sham treatment was dealt with from two angles in this review. Firstly, we were interested to see how often researchers truly cared about the psychological aspect of the sham treatment. This was either determined by us (i.e. we considered that it seemed credible or not) or, better, by the researchers through a post study questionnaire. Secondly, though, we attempted to determine if the sham treatment also had a physiological element to it that resembled the real treatment. Nevertheless, we could not fully exploit this information, because there were too few studies for further analysis.

### Methodological consideration of the reviewed studies

In relation to weaknesses of the reviewed studies, the three most commonly omitted quality items were how authors dealt with missing data, such as their statistical imputation methods for missing data. They also failed to use a blinded statistician, and none performed a post-intervention assessment of the sham. All these omissions could severely compromise the veracity of the data.

Of the eight studies that used a sham procedure, only three were neither psychologically nor physiologically acceptable, and none of these found a significant difference between SM and a sham procedure. However, such significant difference was found for the other five articles. Further, some studies mistakenly used the term “control” to describe a procedure that instead should have been described as an attempt at a sham procedure, as it was stated that they tried to “fool” the subjects.

Unfortunately, one study failed to report between-group results, which made comparison of outcomes impossible for this study.

Generally, however, the quality was quite high, with only one study scoring low. Also, inclusion in a review does not necessarily mean that studies have the same study objectives as the review, which can have a negative effect on the quality scoring without really reflecting badly on the study in question, which may have been perfectly well conducted for its original purposes just not well suited for the reviewers’ specific research questions.

## Conclusion

In asymptomatic subjects, SM does have an effect on PPT but there appears to be no obvious difference in outcome between various types of manual approaches. It was not possible to compare the effect of SM on different spinal regions, as there were not enough data.

### Perspectives

Our finding that “credible” sham studies had “better” results should be challenged in clinical studies on SM as well as in other areas, to see if this is a common moderator of results. Most importantly though is that the clinical significance, such as level of pain reduction or increased pressure pain threshold, should be quantified as well as duration of the effect.

## Abbreviations

NA: Not Applicable
PPT: Pressure Pain Threshold
RCT: Randomized Controlled Trial
SM: Spinal Manipulation
